# Classification of moving coronary calcified plaques based on motion artifacts using convolutional neural networks: a robotic simulating study on influential factors

**DOI:** 10.1186/s12880-021-00680-7

**Published:** 2021-10-19

**Authors:** Magdalena Dobrolińska, Niels van der Werf, Marcel Greuter, Beibei Jiang, Riemer Slart, Xueqian Xie

**Affiliations:** 1 Departments of Radiology, Nuclear Medicine and Molecular Imaging, Medical Imaging Center, University Medical Center Groningen, University of Groningen, PO Box 30.001, 9700 RB Groningen, The Netherlands; 2 Division of Cardiology and Structural Heart Diseases, Medical University of Silesia in Katowice, Ziołowa 45/47, 40-635 Katowice, Poland; 3 Department of Radiology, University Medical Center Utrecht, University of Utrecht, Heidelberglaan 100, 3584 CX Utrecht, The Netherlands; 4 Department of Radiology and Nuclear Medicine, Erasmus Medical Center Rotterdam, Erasmus University, Postbus 2040, 3000 CA Rotterdam, The Netherlands; 5 Radiology Department, Shanghai General Hospital, Shanghai Jiao Tong University School of Medicine, Haining Rd.100, Shanghai, 200080 China; 6 Department of Biomedical Photonic Imaging, Faculty of Science and Technology, University of Twente, Drienerlolaan 5, 7522 NB Enschede, The Netherlands; 7 Department of Robotics and Mechatronics, Faculty of Electrical Engineering, Mathematics and Computer Science, University of Twente, P.O. Box 217, 7500 AE Enschede, The Netherlands

**Keywords:** Artifacts, Artificial intelligence, Imaging phantoms, X-ray computed tomography

## Abstract

**Background:**

Motion artifacts affect the images of coronary calcified plaques. This study utilized convolutional neural networks (CNNs) to classify the motion-contaminated images of moving coronary calcified plaques and to determine the influential factors for the classification performance.

**Methods:**

Two artificial coronary arteries containing four artificial plaques of different densities were placed on a robotic arm in an anthropomorphic thorax phantom. Each artery moved linearly at velocities ranging from 0 to 60 mm/s. CT examinations were performed with four state-of-the-art CT systems. All images were reconstructed with filtered back projection and at least three levels of iterative reconstruction. Each examination was performed at 100%, 80% and 40% radiation dose. Three deep CNN architectures were used for training the classification models. A five-fold cross-validation procedure was applied to validate the models.

**Results:**

The accuracy of the CNN classification was 90.2 ± 3.1%, 90.6 ± 3.5%, and 90.1 ± 3.2% for the artificial plaques using Inception v3, ResNet101 and DenseNet201 CNN architectures, respectively. In the multivariate analysis, higher density and increasing velocity were significantly associated with higher classification accuracy (all *P* < 0.001). The classification accuracy in all three CNN architectures was not affected by CT system, radiation dose or image reconstruction method (all *P* > 0.05).

**Conclusions:**

The CNN achieved a high accuracy of 90% when classifying the motion-contaminated images into the actual category, regardless of different vendors, velocities, radiation doses, and reconstruction algorithms, which indicates the potential value of using a CNN to correct calcium scores.

**Supplementary Information:**

The online version contains supplementary material available at 10.1186/s12880-021-00680-7.

## Highlights

1. CNN achieved a high accuracy of 90% for classifying the motion-contaminated images into the actual category.
2. The classification accuracy increases at higher velocity and higher CT density.
3. CT system, radiation dose and image reconstruction kernel do not influence the classification accuracy.

## Background

Noninvasive assessment of coronary artery disease (CAD) has gained substantial interest [1], due to large number of global deaths [2]. With the introduction of CT, the burden of coronary atherosclerosis can be expressed as a coronary artery calcium (CAC) score, generally expressed as the Agatston score, which is a strong independent predictor of coronary events in intermediate-risk asymptomatic patients [3–5]. Traditionally, the Agatston score is obtained using ECG-triggered non-contrast CT [6].

In the US, almost 7.1 million non-ECG-triggered chest CT scans are performed each year [7, 8]. Because non-ECG-triggered CT demonstrated comparable results in CAC detection to ECG-triggered CT, these scans also have the potential to assess the risk of CAD [9]. Recently, the Society of Cardiovascular CT and the Society of Thoracic Radiology recommended a CAC evaluation of every non-ECG-triggered chest CT examination as a Class I indication [10].

Whether using ECG-triggered cardiac or non-triggered chest scans, an accompanied limitation is the presence of motion artifacts, which considerably decreases the accuracy of CAC detection and quantification [11]. To decrease the motion artifacts in ECG-triggered CT, the temporal resolution should be shorter than 10% of one cardiac cycle time [12]. Even in the relatively low motion phase of 60–70% in the R–R interval, the velocity of the coronary arteries is still approximately 10 mm/s, even at a heart rate < 60 bpm [13]. However, 50% of ECG-triggered cardiac CT scans are performed with a heart rate > 70 bpm [14], which implies a coronary velocity during the CT acquisition phase of at least 30 mm/s. Notwithstanding, the influence of motion is even greater in a non-ECG-triggered chest CT, where coronary motion is up to 60 mm/s.

In a recent review, Waltz et al. concluded that the convolutional neural network (CNN) has expanded the role of automatic detection and measurement of CAC [15]. Šprem et al. proposed a CNN-based method to identify calcified plaques in-vivo that were severely affected by cardiac motion and reached an accuracy of 85.2% [16]. In a multicenter study, Eng et al. showed sensitivities of 71–94% and positive predictive values in the range of 88–100% to detect CAC on non-triggered chest CT [17]. Because motion artifacts are inevitable in the evaluation of coronary calcification, researchers have started to use CNN to alleviate motion artifacts and improve the robustness of CAC scoring. In an ex-vivo experimental study, Zhang et al. used CNN to correct coronary calcium scores and largely reduced Agatston score variations from 38 to 3.7% [18]. However, the generalization ability of CNN for motion artifact recognition heavily depends on a variety of influential factors, besides coronary motion artifacts, also on other technical factors such as CT vendor, radiation dose, and reconstruction kernel.

Before applying a CNN to correct CAC scores in clinical practice in the future, we first conducted an experimental study to simulate motion artifacts of moving coronary calcified plaques using a coronary artery chest phantom, and second, established three CNN architectures to classify the motion-contaminated images of moving coronary calcified plaques, and determined the influential factors for their classification performance. The current study is a first step towards patient-specific motion artifact recognition which could be used to correct CAC scores in the future.

## Methods

**Fig. 1:**
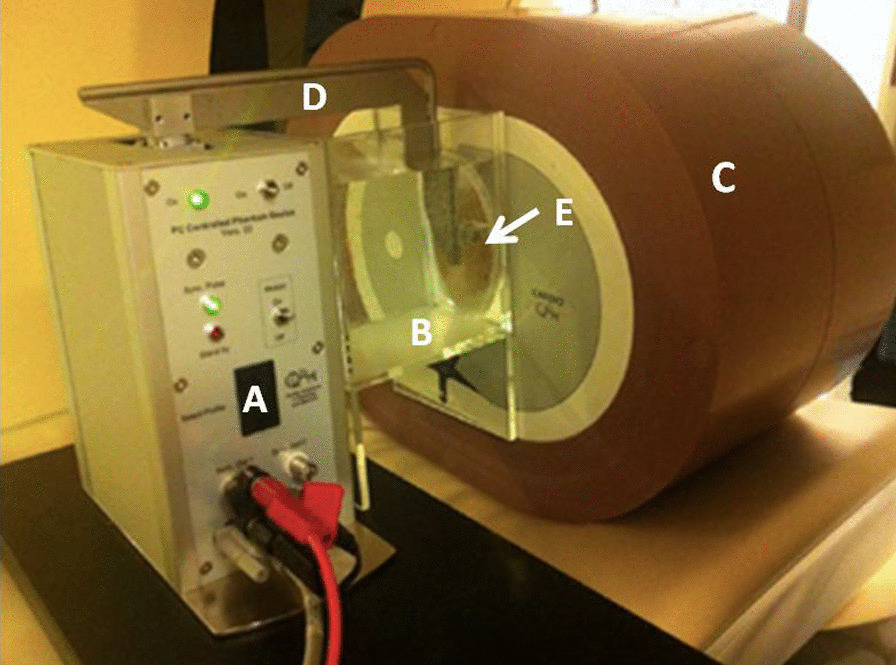
The moving robotic arm with a thoracic phantom. The thoracic phantom includes **a** a computer-controlled motion unit, **b** a water container, **c** a thoracic phantom, **d** a lever, and **e** an artificial coronary artery

Artificial coronary arteries containing cylindrical calcifications moved inside a water container, which was placed at the center of an anthropomorphic chest phantom (QRM-Chest, QRM, Moehrendorf, Germany) (Fig. 1). Inside a shell of tissue-equivalent material, this phantom contained a spine insert and artificial lungs. To mimic an average patient, an extension ring of fat-equivalent material was placed around the phantom to increase the outer dimension to 400 × 300 mm (QRM Extension Ring L, QRM). Movement of the arteries inside the phantom was performed by a computer-controlled lever (QRM-Sim2D, QRM). In total, 7 velocities were assessed for the current study. Each velocity was constant during the scan phase. The used velocities ranged from 0 to 60 mm/s with an increment of 10 mm/s. The movement was in the horizontal *x*-direction, perpendicular to the scan direction. To acquire data during the linear motion of the artificial calcifications, the ECG-trigger of the robotic arm was used, and scans were performed at 60% of the artificial R–R interval. Two artificial coronary arteries were used, each containing two calcium hydroxyapatite (HA) calcifications of equal dimensions: diameter 5.0 ± 0.1 mm and length 10.0 ± 0.1 mm. One artificial coronary artery contained two calcifications with densities of 196 ± 3 and 380 ± 2 mgHA/cm^3^ (physical mass score equal 38 mg, 74 mg respectively), whereas the other contained two calcifications with densities of 408 ± 2 and 800 ± 2 mgHA/cm^3^ (physical mass score equal 80 mg, 157 mg respectively). This corresponded to one low, two medium, and one high coronary plaque burden, respectively (Additional file 1: Table S1).

### CT imaging

Thoracic CT examinations were performed using four CT systems (CT 750 HD, GE Healthcare; Brilliance iCT, Philips Healthcare; Somatom Definition Flash, Siemens Healthineers and Aquilion One, Canon Medical Systems). In the remainder of this paper, the four CT systems are denoted as CT-A to CT-D, respectively (Additional file 1: Table S2). All images were reconstructed using filtered back projection (FBP) and three levels of iterative reconstruction (IR). Each examination was performed at a clinical radiation dose. Subsequently, the radiation dose was reduced by 40% and 80% [19]. Each combination of acquisition settings was repeated five times on every CT scan. In between each scan, the phantom was randomly translated by 2 mm.

### Cross-validation

Since the heterogeneity of the images mainly originated from different vendors of CT, as well as different acquisition protocols, including dose levels and image reconstruction kernels, we conducted a *k*-fold cross-validation across each of these variables. K-fold cross-validation is a widely used resampling procedure to evaluate machine learning models [20]. The *k* parameter of this process represents the number of groups into which all images are divided. In this study, 4 CT systems were used. In the 4 deep learning processes, CT images of 3 CT systems were selected as the training dataset, and the images of the remaining CT were used as the test dataset. In the same way, we performed a threefold cross-validation for dose level and a fourfold cross-validation for image reconstruction kernel. The classification performance of the model is the average performance of these cross-validations.

### Data preparation and image processing

The CT images were categorized into four classes, each corresponding to one calcified plaque, and they included images with different vendors, velocities, radiation doses, and reconstruction algorithms. We remapped the images using a mediastinum window setting (window width 350HU and window level 40HU) to make the images optimal for observation. After defining the calcified plaque manually in CT images, the patch images of calcified plaques were automatically cropped by an in-house developed script based on imcrop function of the Image Processing Toolbox (MATLAB R2020b, MathWorks) and resized to 299 × 299.

Before training the CNN, we first augmented the images using an embedded function to increase the number of training images. The data augmentation was achieved by performing geometric transformations in order to train a robust model, which was invariant to such transformations. Each image was randomly rotated from 0 to 359 degrees, zoomed on with a random aspect ratio ranging from 0.9 to 1.1, translocated from − 30 to 30 pixels, and flipped vertically and/or horizontally with a probability of 0.5. In this way, each original image was augmented to 30 images, resulting in 4 (plaques) × 4 (vendors) × 7 (velocities) × 3 (doses) × 4 (kernels) × 5 (repetitions) × 30 (augmentation) = 201,600 images in total.

### Training algorithm and environment

Three deep CNN architectures were used, i.e., Inception v3, ResNet101, and DenseNet201 (Additional file 1: Tables S3–S5), which are representative in deep learning. The network architectures consisted of 316, 347, and 709 layers, respectively. We adopted the inception and residual architectures because they increase the accuracy by using efficient and deep networks compared to the previous serial CNN architectures [21, 22]. The DenseNet architecture is a logical extension that optimizes the networks by connecting each layer to every other layer in a feed-forward fashion to strengthen feature propagation [23].

We replaced the original classifier layer consisting of 1000 categories by adapting it to the ImageNet dataset with four categories and subsequently fine-tuned the parameters with our training dataset using a back-propagation method across all layers to optimize the networks. The mini-batch sizes were 60, 50, and 25 for these three CNN architectures, respectively, depending on the graphical memory footprints of the computer. The number of training epochs was 15 for the three models, at which point the training accuracy was close to the upper limit. The training dataset was shuffled between each epoch. We used stochastic gradient descent with momentum for the mini-batch gradient descent, which is an iterative method to minimize the result of the loss function of the CNN architectures; thus, it can be used to find suitable and optimized values of network parameters. All layers of the network were fine-tuned using the same global learning rate of 0.0003.

We performed the training and testing procedure using the Deep Learning Toolbox (MATLAB R2020b, MathWorks). The program was implemented on a workstation with a graphics processing unit (RTX 2080Ti, Nvidia).

### Inference method

The CNN inference result for each image of calcified plaque consisted of a one-dimensional numeric series with a length of four, representing the matching probability of the four classifications. Since the length of each artificial calcified plaque was 10 mm, it encompassed at least 3 CT slices at a slice thickness of 3 mm. The three central slices of each calcification were selected manually by a radiologist with 20 years of experience in cardiac imaging. In the test dataset, the average matching probability of these three images was considered as the matching probability of this plaque. Among the four matching probabilities for the four calcifications, the classification of the highest matching probability was considered as the CNN’s classification category.

### Statistics

The ground truth of the CNN algorithms was the physical artificial calcified plaque. It was considered as true positive if the CNNs correctly classified the motion-contaminated images into the actual plaque, that had generated blurred images. We evaluated the classification accuracy and F1 score to represent CNN’s classification performance. The F1 score is a weighted average of the precision and recall, where an F1 score reaches its best value at 1 and worst at 0. Precision, also called the positive predictive value, is the proportion of positive results that truly are positive. Recall is also called sensitivity.

The ROC curve of the CNN’s classification of the calcified plaques with motion artifacts and the area under the curve (AUC) were calculated to access the CNN’s classification performance. The CNN’s matching probability was used as an index variable and the CNN’s classification correctness was used as a reference variable. A multivariate linear regression model was used to synthesize the results of three CNN models [24].

The association between the correctness and potentially associated factors (plaque density, CT vendor, motion velocity, dose level, and reconstruction method) was evaluated using Spearman’s correlation coefficients. Because the classification accuracy was analyzed simultaneously with other variables (density, CT vendor, velocity, dose, and reconstruction algorithm), multivariate analysis was also used to access the association between the correctness and these factors.

The normally distributed outcome parameters are given as the mean values with standard deviations, and non-normally distributed parameters were given as median values with 95% confidence intervals. A *p* value < 0.05 was considered statistically significant. Statistical analyses were performed using a software package (MedCalc 15.8, MedCalc Software).

## Results

### Subjective observation

**Fig. 2:**
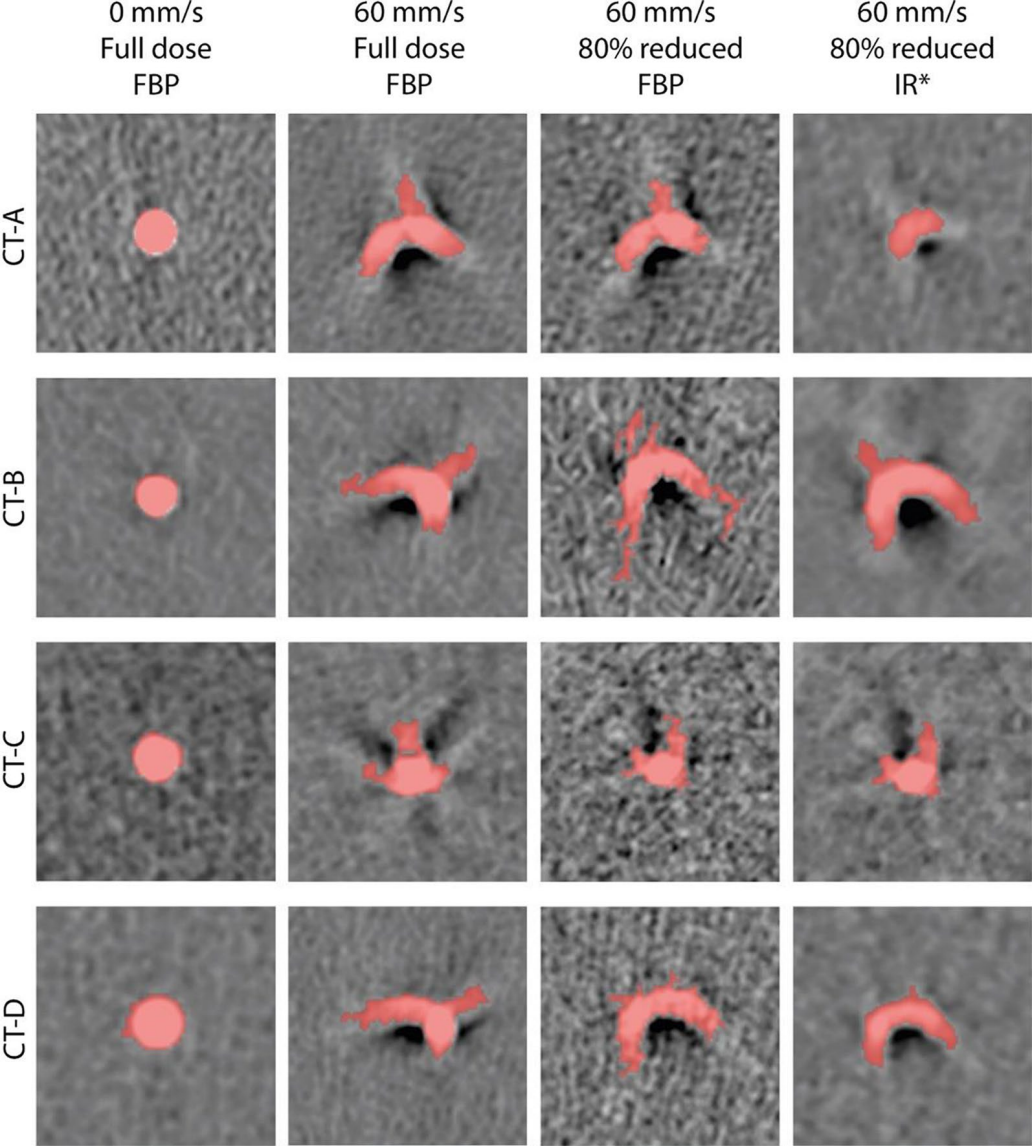
Representative images of the motion artifacts for all CT systems at all velocities, clinical full radiation dose and FBP. Window center was 90 HU and window width was 750 HU. *For each CT system. *FBP* filtered back projection;

**Fig. 3:**
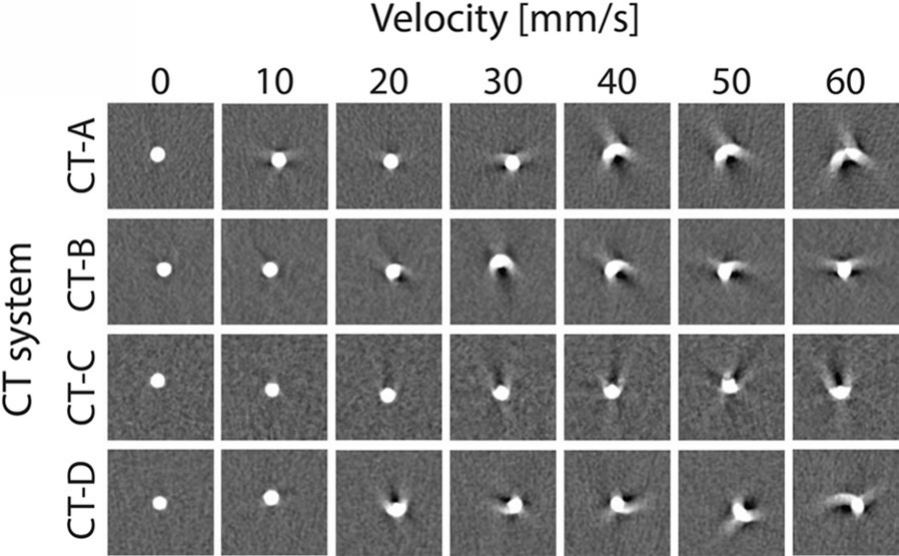
Representative images of the motion artifacts for all CT systems at the minimum and maximum velocity, clinical full and 80% reduced radiation dose and FBP and IR. Window center was 90 HU and window level was 750 HU. *For each CT system, the highest available level of IR was used. *FBP* filtered back projection, *IR* iterative reconstruction

The representative motion artifacts for the different CT systems, velocities, radiation doses, and reconstruction methods are shown in Figs. 2 and 3. Increased blurring was observed as the velocity increased and radiation dose decreased. For each CT scanner, the use of IR resulted in decreased blurring. The smallest blurring was presented by CT-C.

### Cross-validation

In the fourfold cross-validation on CT system, the accuracy and F1 scores of CNN in the classification of 4 artificial plaques were high but variable (Additional file 1: Tables S6–S9). The accuracies ranged from 84.8 to 95.3%, 83.8 to 95.7%, and 83.4 to 96.8% for Inception v3, ResNet101, and DenseNet201, respectively. The F1 scores ranged from 0.849 to 0.966, 0.859 to 0.969, and 0.883 to 0.969, respectively.

In the threefold cross-validation on dose level, the accuracy and F1 scores of CNN were also high but variable. The accuracies ranged from 86.1 to 93.7%, 83.2 to 94.6%, and 85.2 to 94.4%, and the F1 scores ranged from 0.871 to 0.959, 0.881 to 0.967, 0.886 to 0.956. In the fourfold cross-validation on reconstruction kernel, the accuracies ranged from 85.9 to 96.1%, 84.9 to 95.9%, 83.3 to 96.0%, and the F1 scores ranged from 0.893 to 0.963, 0.871 to 0.963, and 0.867 to 0.966.

### Overall agreement between actual and predicted labels

**Fig. 4:**
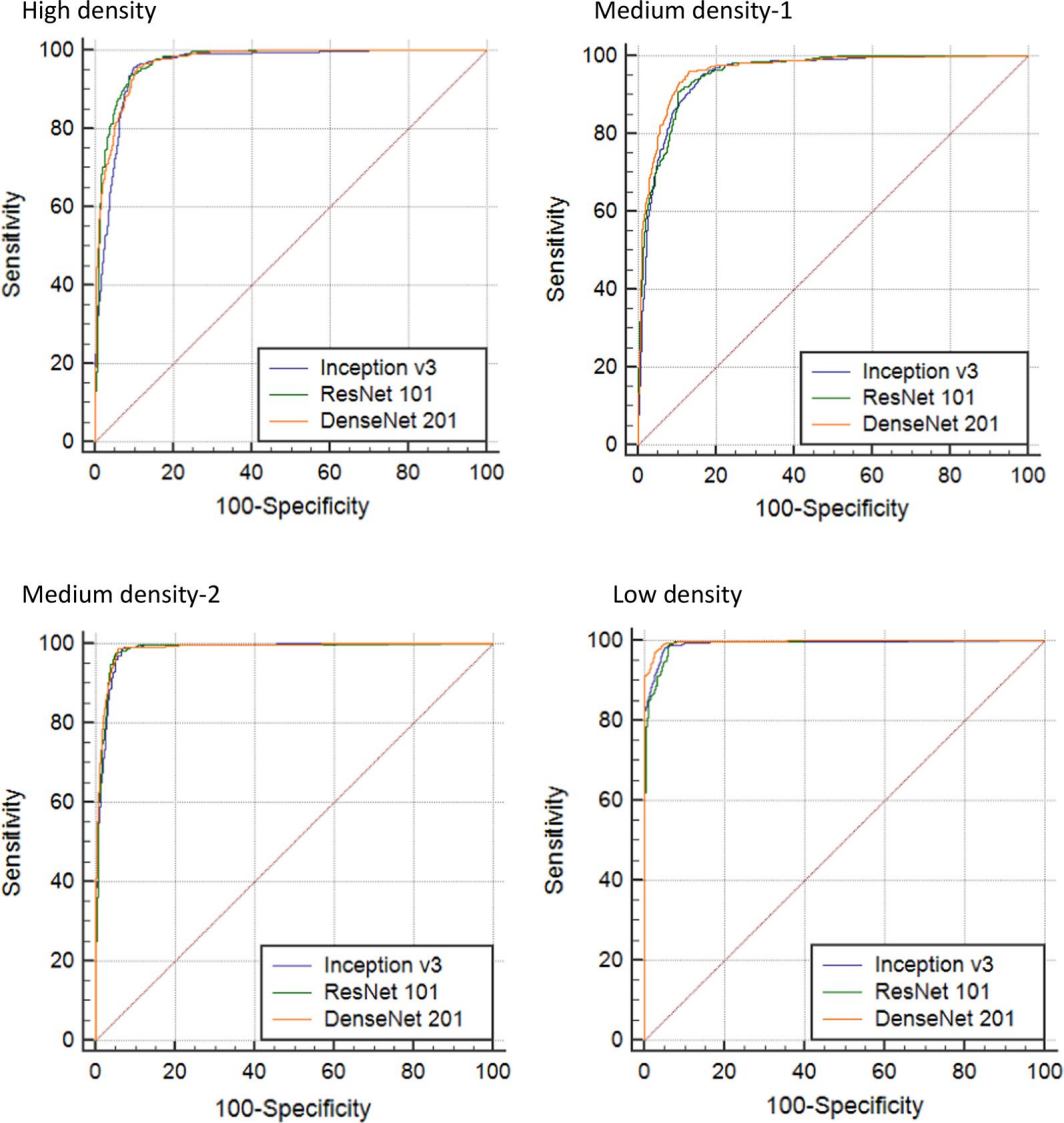
Receiver operating characteristic curves of convolutional neural network’s classification on calcified plaques with motion artifacts

The overall accuracy of the CNN classification for all four artificial plaques was 90.2 ± 3.1%, 90.6 ± 3.5%, and 90.1 ± 3.2% for inception v3, ResNet101 and DenseNet201 CNN, respectively. The low-density plaque showed the highest accuracy of 93.3 ± 1.6%, 92.4 ± 2.8% and 92.7 ± 2.8%, respectively; and F1 scores of 0.950 ± 0.012, 0.947 ± 0.020 and 0.945 ± 0.019, respectively (Table 1). The AUCs were 0.982 (95% CI 0.976–0.986), 0.981 (0.974–0.992) and 0.986 (0.982–0.994), respectively (Table 2 and Fig. 4). The medium-density-1 plaque showed the lowest accuracy of 88.0 ± 3.0%, 87.1 ± 2.3% and 87.7 ± 2.9%, respectively; F1 scores of 0.901 ± 0.022, 0.896 ± 0.020 and 0.897 ± 0.021, respectively; and AUCs of 0.951 (0.970–0.962), 0.955 (0.943–0.981) and 0.962 (0.951–0.972), respectively. An ensemble of three CNN models by a multivariate linear regression model slightly increased the AUC to 0.990 (0.982–0.998).

**Table 1:** Classification accuracy and F1 scores of Inception v3, ResNet101 and DenseNet201 convolutional neural networks on calcified plaques with motion artifacts of four densities

Plaque density	Inception v3		ResNet101		DenseNet201	
	Accuracy	F1 score	Accuracy	F1 score	Accuracy	F1 score
High	88.8 ± 2.3%	0.917 ± 0.024	90.2 ± 2.8%	0.922 ± 0.027	89.3 ± 2.9%	0.919 ± 0.023
Medium-1	88.0 ± 3.0%	0.901 ± 0.022	87.1 ± 2.3%	0.896 ± 0.020	87.7 ± 2.9%	0.897 ± 0.021
Medium-2	90.7 ± 2.5%	0.939 ± 0.024	92.9 ± 2.9%	0.942 ± 0.028	90.7 ± 2.0%	0.937 ± 0.018
Low	93.3 ± 1.6%	0.950 ± 0.012	92.4 ± 2.8%	0.947 ± 0.020	92.7 ± 2.8%	0.945 ± 0.019

Variables are displayed as mean ± standard deviation

**Table 2:** The area under receiver operating characteristic curves of convolutional neural network’s classification on calcified plaques with motion artifacts

Plaque density	Inception v3	ResNet101	DenseNet201
High	0.952 (0.939–0.964)	0.972 (0.962–0.980)	0.970 (0.960–0.978)
Medium-1	0.951 (0.939–0.962)	0.955 (0.943–0.965)	0.962 (0.951–0.972)
Medium-2	0.980 (0.970–0.989)	0.974 (0.969–0.981)	0.976 (0.970–0.982)
Low	0.982 (0.976–0.986)	0.981 (0.974–0.992)	0.986 (0.982–0.994)

The data is expressed as area under the curve (95% confidence interval)

**Fig. 5:**
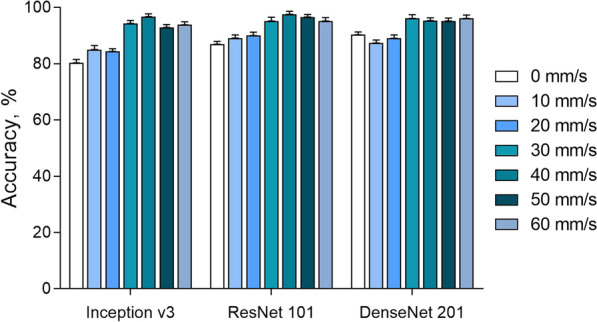
Classification accuracy of Inception v3, ResNet101, and DenseNet201 convolutional neural network on calcified plaques with motion artifacts in the velocity from 0 to 60 mm/s

With respect to different CT vendors, the accuracy was similar, and ranged from 89.8 ± 2.7% to 91.8 ± 0.8%, 88.0 ± 5.3% to 92.2 ± 2.3%, and 89.3 ± 5.6% to 92.0 ± 1.8% for inception v3, ResNet101 and DenseNet201 CNN, respectively (Table 3). Regarding the velocity, the plaques at rest showed relatively lower accuracies of 80.3 ± 1.3%, 85.4 ± 1.4%, and 89.2 ± 1.2%, respectively (Fig. 5). When the velocity increased to 60 mm/s, the accuracy increased to 93.8 ± 1.3%, 95.1 ± 1.6%, and 96.1 ± 1.5%, respectively.

**Table 3:** Classification accuracy of Inception v3, ResNet101 and DenseNet201 convolutional neural network on calcified plaques with motion artifacts on four CT systems

	Inception v3	ResNet101	DenseNet201
CT-A	90.2 ± 3.1%	92.2 ± 2.3%	92.0 ± 1.8%
CT-B	89.8 ± 2.7%	88.0 ± 5.3%	89.3 ± 5.6%
CT-C	91.0 ± 2.8%	90.9 ± 2.6%	90.7 ± 2.4%
CT-D	91.8 ± 0.8%	91.2 ± 3.6%	91.1 ± 2.6%

Variables are displayed as mean ± standard deviation

### Univariate association between influencing factors and CNN’s classification

Lower plaque density and increasing velocity were significantly associated with higher classification accuracy in all three CNN architectures (all *P* < 0.001). However, other factors (CT system, radiation dose and reconstruction method) had no significant influence on the accuracy of CNN classification (all *P* > 0.05) (Table 4).

**Table 4:** Spearman’s correlation coefficients (rho) for the univariate association between influencing factors and convolutional neural network’s classification on calcified plaques with motion artifacts

	Inception v3		ResNet101		DenseNet201	
	rho (95% CI)	*p* value	rho (95% CI)	*p* value	rho (95% CI)	*p* value
Density	0.139 (0.083, 0.195)	< 0.001	0.199 (0.061, 0.337)	< 0.001	0.194 (0.074, 0.314)	< 0.001
CT vendor	0.031 (− 0.017, 0.079)	0.249	− 0.049 (− 0.151, 0.053)	0.210	− 0.102 (− 0.192, − 0.012)	0.107
Velocity	0.191 (0.105, 0.277)	< 0.001	0.169 (0.029, 0.309)	< 0.001	0.163 (0.024, 0.302)	< 0.001
Dose	− 0.028 (− 0.081, 0.025)	0.518	0.028 (− 0.052, 0.108)	0.477	0.046 (− 0.010, 0.102)	0.239
Reconstruction	0.019 (− 0.031, 0.069)	0.312	0.118 (0.030, 0.206)	0.230	0.134 (0.023, 0.245)	0.222

High, medium-1, medium-2, and low-density plaque were coded as 1–4, respectively, four CT systems (CT-A to CT-D) as 1–4; velocities from 0 to 60 mm/s coded as 0–6; dose level 40%, 80% and full dose coded as 1–3; recon method FBP, IR1 to IR3 coded as 1–4

*FBP* filtered back projection, *IR* iterative reconstruction

### Multivariate analysis for factors associated with CNN’s classification

In the multivariate analysis, a higher density and an increasing velocity were significantly associated with a higher classification accuracy for all three CNN architectures (all *P* < 0.001). The classification accuracy for all three CNN architectures was not affected by CT system, radiation dose or reconstruction method (all *P* > 0.05) (Table 5).

**Table 5:** Multivariate analysis for the influencing factors associated with CNN’s classification on calcified plaques with motion artifacts

	Inception v3		ResNet101		DenseNet201	
	Coefficient	*p* value	Coefficient	*p* value	Coefficient	*p* value
Density	0.033	< 0.001	0.024	< 0.001	0.319	< 0.001
CT vendor	0.012	0.147	− 0.025	0.091	− 0.038	0.102
Velocity	0.027	< 0.001	0.017	< 0.001	0.015	< 0.001
Dose	− 0.009	0.601	− 0.011	0.159	0.002	0.779
Reconstruction	0.009	0.126	0.010	0.112	0.012	0.099

High, medium-1, medium-2, and low-density plaque were coded as 1–4, respectively, four CT systems (CT-A to CT-D) as 1–4; velocities from 0 to 60 mm/s coded as 0–6; dose level 40%, 80% and full dose coded as 1–3; recon method FBP, IR1 to IR3 coded as 1–4

*FBP* filtered back projection, *IR* iterative reconstruction

## Discussion

In this experimental study, we applied three widely-used deep CNN architectures in medical image analysis [25–27] to classify the CT images of calcified coronary plaques with motion artifacts into the correct category with a high accuracy, regardless of different CT vendors, velocities, radiation doses, and image reconstruction algorithms. The overall classification accuracy of these CNNs reached 90%.

ECG-triggered CT and non-ECG-triggered CT are vulnerable to a great variety of artifacts. The most common cause of artifacts is motion [28]. Typical motion artifacts are blurring, ghosting, or windmills, which influence accurate CAC classification [29]. The severity of the motion artifacts depends not only on the heart rate and temporal resolution of the CT scanner but also on the specific coronary artery. The mean velocity of the right coronary artery is significantly higher than that of the left anterior descending and circumflex coronary artery [30]. In our study, all the CNN architectures resulted in higher classification accuracy at higher velocities. In contrast to the plaques at rest, which showed no motion artifacts, the plaques showed spatially more dispersed motion artifacts caused by increasing velocities (Fig. 2). The magnitude of the artifact means that more image features that can be recognized and used for classification by a CNN.

Motion artifacts appear when the temporal resolution is insufficient to warrant data acquisition during the time the coronary arteries exhibit the least motion. The temporal resolution can be improved by a shorter gantry rotation time, or dedicated acquisition and reconstruction protocols [31]. It has been demonstrated that plaque classification also depends on the density and size, where relatively small and soft calcified plaques may remain undetected [32]. Van der Werf et al. found that at an increased heart rate, the Agatston scores of low-density calcified plaques were similar to the reference scores but that the Agatston scores of medium- and high-density calcified plaques were increased by up to 50% [11]. In our study, all CNN architectures gained the highest classification accuracy for plaques with low density.

Another difficulty that a CNN encounters during plaque motion artifact classification is increased noise in low-dose CT scans. Currently, IR algorithms can be used for CT image reconstruction to reduce the noise in low-dose CT scans [33, 34]. In a phantom study, increased IR levels resulted in decreased CAC scores [19]. In clinical practice for CAC scoring, a soft kernel is used in which the noise is less prominent. However, in non-contrast and non-ECG-triggered CT scans performed for other diagnostic purposes, generally, sharper kernels are used. Recently, it was found that if a CNN is trained on both soft and sharp kernels, the accuracy of the CNN in CAC scoring is similar to that of soft kernel CT scans [35]. In our study, both reconstruction methods did not influence the accuracy of all three CNN architectures. Furthermore, a lower radiation dose also did not affect the CNN’s classification.

So far, for CAC classification in non-contrast and ECG-triggered CT scans, different machine-learning-based techniques have been studied to improve the diagnostic management in everyday practice. Most of these methods focused on calcium detection and coronary artery calcium scoring [35–37]. In contrast, the method presented in the current study was based on training CNNs on motion artifacts, which are one of the main limitations in accurate CAC score measurement. Motion artifact recognition methods based on motion correction algorithms have shown their value in the image improvement of coronary CT angiography (CCTA) [38]. Furthermore, a CNN was used to estimate the artifact motion vectors from CCTA images and the method improved the quality and showed potential to be applied in clinical practice [39]. In our study, CNNs were solely used for motion artifact recognition from non-contrast and ECG-triggered CT scans to enhance the assessment of CAC scores in the future. Since phantoms were used for the current study, the increase of the motion artifacts as heart rate increased was known. This led to enhanced accuracy of CNNs since motion artifact features increased as the heart rate increased. Although this is of course not feasible in daily practice, our results indicated that a CNN may play an important role in clinical coronary calcium classification in the near future.

The main limitation of this study is the linear movement of artificial arteries, their relatively high diameters, and the regular shape of calcifications, which do not reflect the movement and shape of coronary arteries in vivo. Additionally, a variety of body habitus were not also included.

In this experimental study, the CNN achieved a high accuracy of 90% at classifying motion-contaminated images into the actual category, regardless of different influential technical factors. This study validated the first step towards patient-specific motion artifacts recognition which could be used to correct CAC scores in the future. Interestingly, the classification accuracy increased at higher velocities because the magnitude of the artifact had more image features that may be recognized and used for classification by a CNN, which inspires us to optimize the calcium score correction method in cases of heavily motion-contaminated images.

## Supplementary Information

**Additional file 1**. Calcified plaques, CT imaging protocol, and neural network structure in this study.

## Data Availability

The code is available for scientific purpose, upon request to the corresponding author.

## Abbreviations

CAC: Coronary artery calcium
CAD: Coronary artery disease
CNN: Convolutional neural network
FBP: Filtered back projection
IR: Iterative reconstruction
JPEG: Joint Photographic Experts Group
