# Supplementary material for: Classification of moving coronary calcified plaques based on motion artifacts using convolutional neural networks: a robotic simulating study on influential factors
Source: BMC Med Imaging. 2021 Oct 19;21:151. doi: 10.1186/s12880-021-00680-7 (PMC8524892; doi:10.1186/s12880-021-00680-7)
Supplement: Supplementary file 1 — Additional file 1. Calcified plaques, CT imaging protocol, and neural network structure in this study. [file 12880_2021_680_MOESM1_ESM.pdf]

Supplementary Table A.1. Physical mass and corresponding mass scores for the artificial calcified plaques at rest and filtered back projection reconstruction.

Plaque density	Physical mass, mg	CT system	Mass score, mg
**High**	157	CT-A	125 (108 – 138)
		CT-B	165 (161 – 175)
		CT-C	131 (128 – 136)
		CT-D	188 (186 – 191)
**Medium-1**	80	CT-A	70 (60 – 78)
		CT-B	76 (75 – 79)
		CT-C	62 (59 – 65)
		CT-D	86 (80 – 94)
**Medium-2**	74	CT-A	58 (54 – 62)
		CT-B	63 (59 – 68)
		CT-C	49 (47 – 53)
		CT-D	69 (66 – 72)
**Low**	38	CT-A	23 (20 – 26)
		CT-B	25 (22 – 26)
		CT-C	20 (16 – 22)
		CT-D	26 (23 – 29)

Note. The mass scores are expressed as median and range.

Supplementary Table A.2. CT acquisition protocol and image reconstruction parameters

	CT-A (CT 750 HD, GE Healthcare)	CT-B (Brilliance iCT, Philips Healthcare)	CT-C (Somatom Definition Flash, Siemens Healthineers)	CT-D (Aquilion One, Canon Medical Systems)
Tube voltage	120kV	120kV	120kV	120kV
Tube charge per rotation (mA)	500	185	285	230
Collimation (mm)	64 × 0.625	128 × 0.625	128 × 0.6	320 × 0.5
Rotation time (s)	0.35	0.27	0.28	0.35
Temporal resolution[a] (ms)	175	135	75	175
Kernel	Standard	XCA	B35f	FC12
Slice thickness	2.5	3.0	3.0	3.0
Slice increment	2.5	3.0	3.0	3.0
$CTDI_{vol}$ (mGy)	10.6	3.2	2.8	6.5
Software	Smartscore 4.0	Heartbeat-CS	Syngo	Vitrea FX 6.5.0

Supplementary Table A.3. Architecture of Inception v3 convolutional neural network

Layer type	Patch size/stride	Input size
conv	3×3/2	299×299×3
conv	3×3/1	149×149×32
Conv padded	3×3/1	147×147×32
Pool	3×3/2	147×147×64
conv	3×3/1	73×73×64
conv	3×3/2	71×71×80
conv	3×3/1	35×35×192
3×Inception	Mixed	35×35×288
5×Inception	Mixed	17×17×768
2×Inception	Mixed	8×8×1280
Pool	8×8	8×8×2048
Linear	Logits	1×1×2048
Softmax	Classifier	1×1×4

Supplementary Table A.4. Architecture of ResNet 101 convolutional neural network

Layer type	Patch size/stride	Output size
conv1	7×7, 64, stride 2	112×112
conv2 x	3×3 max pool, stride 2	56×56
	$\begin{bmatrix} 1\times 1, & 64 \\ 3\times 3, & 64 \\ 1\times 1, & 256 \end{bmatrix}$×3	
conv3 x	$\begin{bmatrix} 1\times 1, & 128 \\ 3\times 3, & 128 \\ 1\times 1, & 512 \end{bmatrix}$×4	28×28
conv4 x	$\begin{bmatrix} 1\times 1, & 256 \\ 3\times 3, & 256 \\ 1\times 1, & 1024 \end{bmatrix}$×23	14×14
conv5 x	$\begin{bmatrix} 1\times 1, & 512 \\ 3\times 3, & 512 \\ 1\times 1, & 2048 \end{bmatrix}$×3	7×7
	average pool, 1000-d fc, softmax	1×1

Supplementary Table A.5. Architecture of DenseNet 201 convolutional neural network

Layer type	Patch size/stride	Output size
Convolution	7 × 7 conv, stride 2	112 × 112
Pooling	3 × 3 max pool, stride 2	56 × 56
Dense Block (1)	$\begin{bmatrix} 1 \times 1 & \text{conv} \\ 3 \times 3 & \text{conv} \end{bmatrix} \times 6$	56 × 56
Transition Layer (1)	1 × 1 conv	56 × 56
	2 × 2 average pool, stride 2	28 × 28
Dense Block (2)	$\begin{bmatrix} 1 \times 1 & \text{conv} \\ 3 \times 3 & \text{conv} \end{bmatrix} \times 12$	28 × 28
Transition Layer (2)	1 × 1 conv	28 × 28
	2 × 2 average pool, stride 2	14 × 14
Dense Block (3)	$\begin{bmatrix} 1 \times 1 & \text{conv} \\ 3 \times 3 & \text{conv} \end{bmatrix} \times 48$	14 × 14
Transition Layer (3)	1 × 1 conv	14 × 14
	2 × 2 average pool, stride 2	7 × 7
Dense Block (4)	$\begin{bmatrix} 1 \times 1 & \text{conv} \\ 3 \times 3 & \text{conv} \end{bmatrix} \times 32$	7 × 7
Classification Layer	7 × 7 global average pool	1 × 1
	1000D fully-connected, softmax	
